# Late recurrence of Burkitt’s lymphoma in the jaw: numb chin syndrome as the only symptom

**DOI:** 10.4322/acr.2020.218

**Published:** 2020-12-08

**Authors:** Bernar Monteiro Benites, Wanessa Miranda-Silva, André Caroli Rocha, Ula Lindoso Passos, Felipe Paiva Fonseca, Celso Arrais Rodrigues da Silva, Eduardo Rodrigues Fregnani

**Affiliations:** 1 Hospital Sírio-Libanês, Serviço de Medicina Bucal, São Paulo, SP, Brasil; 2 Universidade de São Paulo (USP), Faculdade de Medicina, Hospital das Clínicas, São Paulo, SP, Brasil; 3 Hospital Sírio-Libanês, Serviço de Radiologia, São Paulo, SP, Brasil; 4 Universidade de Minas Gerais (UFMG), Faculdade de Odontologia, Departamento de Patologia Oral, Belo Horizonte, MG, Brasil; 5 Hospital Sírio-Libanês, Unidade de Transplante de Medula Óssea, São Paulo, SP, Brasil

**Keywords:** Burkitt Lymphoma, Recurrence, Hypesthesia, B-lymphocytes

## Abstract

The Numb Chin Syndrome (NCS) is defined as facial and oral numbness restricted to the mental nerve’s distribution involving the lower lip, skin of the chin, or gingiva of the lower anterior teeth. Hypoesthesia can occur unilaterally or bilaterally. Although this syndrome is rare, its importance is related to the fact that it represents the clinical manifestations of malignant diseases. Breast cancer and non-Hodgkin lymphoma are the most common cause of NCS. The patient, a 58-year-old woman, treated for a Burkitt Lymphoma (BL) nine years ago, described a two-week history of change in sensitivity and pain in the chin region, without relief with the use of analgesics. She had no headache, speech disturbance, dysphagia, visual disturbance, or other neurological symptoms. No surgical intervention has been performed recently. The intraoral examination revealed a healthy oral mucosa and a small area adjacent to the right mental nerve region that was uncomfortable to palpation. No changes were found in the bone trabeculae at cone-beam computed tomography. The contrasted magnetic resonance features made it possible to identify a change in the mandibular body extending to the entire right side, coinciding with the patient’s complaint, indicating a probable mandibular medullary invasion. The patient was submitted to a biopsy to rule out a possible recurrence of BL. The microscopic findings were consistent with the diagnosis of BL. The present report described a very unusual presentation of late recurrent BL nine years after the first treatment, which manifested as an NCS.

## INTRODUCTION

The Numb Chin Syndrome (NCS), also called mental nerve neuropathy, is defined as facial and oral numbness restricted to the mental nerve’s distribution involving the lower lip, skin of the chin, or gingiva of the lower anterior teeth. It causes a sensory neuropathy-like unilateral, or bilateral hypoesthesia, paresthesia or anesthesia in the area supplied by the trigeminal nerve’s mental branch. These symptoms reflect the anatomical substrate of this syndrome.^1^ ^,^ ^2^

Despite being a rare clinical manifestation, the importance of this syndrome lies in its frequent association with malignancies.^3^ The most common non-hematologic neoplastic cause of NCS is breast cancer, while the most common hematologic neoplastic cause is non-Hodgkin lymphoma (NHL).^1^

Lymphomas are malignant tumors derived from the lymphoreticular or immune system. BL belongs to the NHL group and is characterized by a very high and diffuse proliferation of B lymphocytes.^2^ ^,^ ^4^

Charles Bell first described NCS in 1988. This syndrome may be the first symptom of diseases arising from different etiologies, including traumatic, vascular, inflammatory, demyelinating, infectious, and neoplastic disorders.^5^ Therefore, the presence of NCS conditions is suspected often enough to warrant a search for these possible etiologies, because, in patients with a history of cancer, NCS often indicates disease recurrence or progression with a poor prognosis.^2^

In the present paper, we present a rare case of late recurrence of BL initially presenting with bilateral NCS.

## CASE REPORT

The patient, a 58-year-old woman, treated for a BL nine years ago, described a two-week history of change in sensitivity and pain in the chin region, which did not relief with the use of analgesics. She had no headache, speech disturbance, dysphagia, visual disturbance, or other neurological symptoms.

At first, she looked for her hematologist-oncologist because of her past medical history of BL. Physical examination revealed complete paresthesia involving the chin and lower lip bilaterally, without any trauma and no evident increase in volume or swelling, only the numbness in the mental region. There was no clinical evidence of palpable regional lymph nodes, and all other systems were found to be clinically normal.

**Figure 1:**
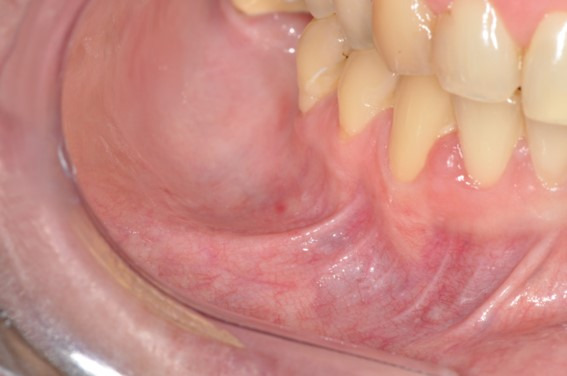
Intraoral examination revealed a healthy oral mucosa.

The patient was referred to the Oral Medicine Department, and the intraoral examination revealed a healthy oral mucosa, no recent tooth extraction, no trauma, and no oral surgery (Figure 1).

**Figure 2:**
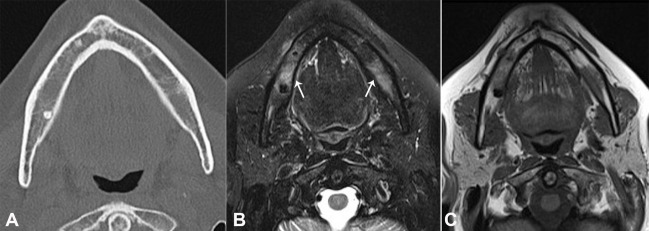
**A** – Computed tomography of the mandible (axial plane) - note a reduction of bone trabeculations in the mandibular body, possibly related to infiltration; **B** and **C** – Axial plane of MRI with bilateral mandibular bone marrow signal alteration (arrows); **B –** T2 high signal tissue near the right mental foramen related to the involvement of the inferior alveolar nerve (arrow); **C** – Confirmation of the image in T1-weighted sequence.

As a result of the patient’s complaint, we identified a small area adjacent to the region of the right mental nerve that was uncomfortable on palpation. No other changes were found by means of cone-beam computed tomography (Figure 2A) and clinical examination. Magnetic resonance features made possible to identify a change in the mandibular body extending to the entire right side (Figures 22C, 33B), coinciding with the clinical complaint, indicating a probable mandibular medullary invasion.

**Figure 3:**
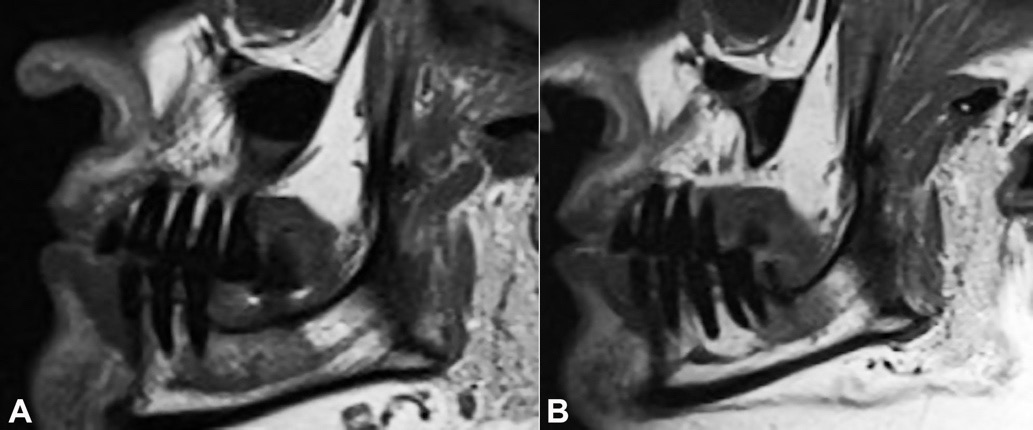
**A** – Left and **B –** right sagittal section of MRI (T1 weighed images). In both images, asymmetry of the mandibular canals is observed, with more evidence on the right side

**Figure 4:**
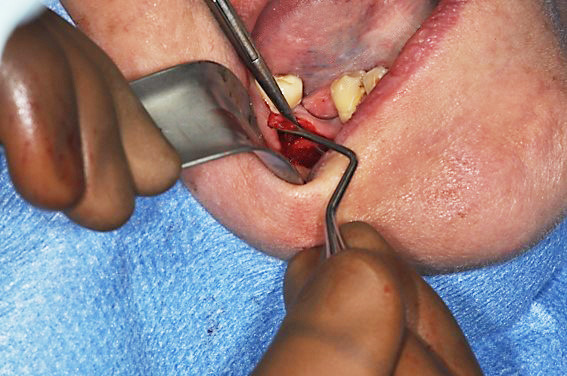
Intraosseous medullar biopsy of the mandible.

At this time, considering the medical history, an intraosseous biopsy (Figure 4) adjacent to the right mental nerve was performed to pursue a possible recurrence of BL.

**Figure 5:**
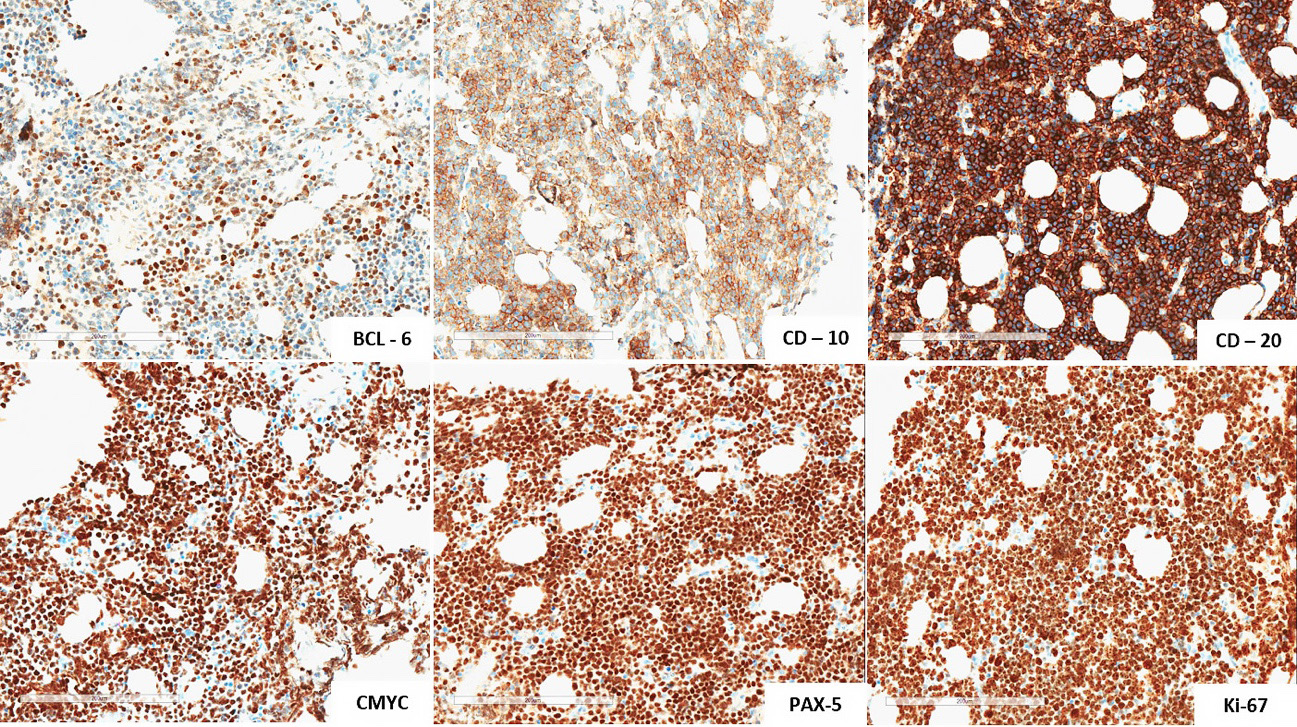
Immunohistochemical profile, revealed positivity for BCL-6, CD-10, CD-20, C-MYC, PAX-5 and strong positivity for Ki-67.

The microscopic analysis of the bone biopsy demonstrated a diffuse proliferation of moderate-sized neoplastic cells, exhibiting hyperchromatic nucleoli, and scant cytoplasm. A focal starry-sky pattern was observed. The immunohistochemical profile (Figure 4) revealed positivity for BCL-6, CD-10, CD-20, C-MYC, PAX-5, and strong positivity for Ki-67, demonstrating the presence of a mature B-cell lineage, consistent with the diagnosis of BL (Figure 5). The *in-situ* hybridization was negative for Epstein-Barr virus. Rescue chemotherapy treatment was initiated to enable an autologous transplant to be performed.

## DISCUSSION

Lymphoma is a heterogeneous malignant disease of the lymphatic system, characterized by a proliferation of lymphoid cells or their precursors. Lymphomas represent the third most common malignancy affecting the oral cavity and jawbones after squamous cell carcinoma and the malignant tumor of the salivary glands.^4^ They can be divided into two large groups: Hodgkin’s lymphoma (HL) and non-Hodgkin’s Lymphoma (NHL).^6^ ^,^ ^7^

Short et al.^8^ identified 145 adults with BL with a median age of 51 years (18-76 years). They defined a cut-off point: early and late relapses were defined as relapse <6 months and ≥6 months from the time of the first remission. In our case, we present an extremely rare and late case. Twelve patients died before initial response assessment, and 2 were unevaluable due to inadequate records, which demonstrated the severity of the disease. Among the 131 patients evaluable for response, 35 patients (27%) had relapsed (n=32) or were refractory (n=3). Ten patients experienced late relapse after the first remission and had a median overall survival of 5.0 months.

The first report of mental neuropathy associated with malignancies was filed in 1963 with the description of 5 cases of patients with metastatic malignant disease, who had NCS as the initial presentation.^1^ ^,^ ^9^ The NCS received this term because it is a sensory neuropathy characterized by hypoesthesia, paresthesia or anesthesia of the area innervated by the mental nerve and its branches.^2^ This nerve has no motor fibers, and NCS is a purely sensory neuropathy.^3^ Furthermore, this can result from mechanical nerve compression caused by osseous involvement of the mandible or nerve damage caused by tumor infiltration along the nerve sheath.^1^

This syndrome has been attributed to benign and malignant conditions,^2^ including lymphoma, acute leukemia, BL, multiple myeloma, Ewing sarcoma, melanoma, breast cancer, prostate cancer, lung cancer, colon cancer, and esophageal cancer.^1^ In a retrospective case series of NCS, the incidence indicated a significant difference between patients with metastatic cancer (23%, 12 of 53) and those with benign diseases (4%, 4 of 104), showing the importance of NCS as a neurological manifestation in cases of cancer and/or metastatic malignancy.^5^

Some cases of NCS may also be the first sign of recurrence or metastasis in patients with a history of malignancy, or sometimes the first manifestation in hematologic malignancies.^2^ Sasaki et al.^1^ reviewed some cases, and the onset of bilateral NCS preceded the diagnosis of the primary tumor in 8 out of the 22 cases. Lu et al.^5^ described a total of 12 patients who complained about oral symptoms that led to the diagnoses of NCS associated with metastatic cancer. Although they are not specific symptoms, dentists and physicians need to take special heed of these symptoms.

This syndrome requires special attention from clinicians in BL cases to avoid misdiagnosis since there are other causes of NCS. A review of literature^1^ ^,^ ^2^ ^,^ ^10^ showed some other etiologies of NCS, including dental or odontogenic processes such as (i) trauma, (ii) infection, (iii) locally invasive tumors, (iv) mandibular osteomyelitis; (v) metastatic neoplasms; (vi) multiple myeloma; (vii) sickle cell disease; (viii) multiple sclerosis; (ix) sarcoidosis; (x) Lyme disease; (xi) temporal arteritis; (xii) diabetic neuropathy; (xii) AIDS; and (xiv) medication-related osteonecrosis of the jaw (MRONJ).

Fortunato et al.^3^ conducted a study from 2010 to 2016 recruiting patients who presented NCS as one of their symptoms, excluding those in whom it depended on a clear odontogenic cause, systemic degenerative diseases or metabolic disorders. As the first symptom, 116 patients presented NCS, but only 29 were included in the study. The family dentist was the first to observe the symptom in the great majority of cases (62%); only 6 patients were referred by an oncologist. In all cases, NCS was unilateral; in 11 cases, it was the first symptom of malignancy; and in only 2 cases (6.8%) the patients had non-Hodgkin Lymphoma.

On the other hand, Sasaki et al.,^1^ conducted a review of bilateral numbness of the lower lip and chin and found 22 cases of bilateral NCS associated with malignancy. The primary disease was BL in 5 cases. Interestingly, as previously reported, the onset of bilateral NCS preceded the diagnosis of the primary tumor in 8 out of the 22 cases. In the remaining 14 cases, the onset of NCS was associated with progression, recurrence, or metastasis of the primary tumor, in a manner similar to that of our case. The above-mentioned review detected more cases of bilateral NCS associated with hematologic malignancies than with solid malignancies. Algahtani et al.,^2^ in their review, believed that this could be explained by the fact that hematologic malignancies infiltrated into the central nervous system more readily than did the solid neoplasms.

In clinical practice, most clinicians do not request panoramic radiography of the jaw to start the investigation. In general, physicians do not start with either computed tomography (CT) or magnetic resonance image (MRI) of the jaw, face, and brain, which fails to place value on the patient's chief complaint. CT imaging of the brain and base of the skull makes it possible to identify lytic lesions involving the inferior alveolar nerve or the mental foramen. Some authors have recommended MRI scanning of the face if the etiology of the mental neuropathy is unclear after routine imaging studies have been performed.^2^

According to the characteristics of each case, there were some possible diagnostic hypotheses to be considered, such as Fortunato et al.^3^ in their patients: osteomyelitis, MRONJ, odontogenic tumor, odontogenic cysts, because local dental causes are most often responsible for NCS. Although there are few case reports in the literature^1^ ^,^ ^2^ ^,^ ^4^ ^,^ ^10^ all studies were in agreement in saying that NCS may be the first sign of recurrence or disease progression in patients with a history of cancer.

In conclusion, the present report described a very unusual presentation of late recurrent BL nine years after the first treatment, which manifested as an NCS.
